# Long non-coding RNAs in Epstein–Barr virus-related cancer

**DOI:** 10.1186/s12935-021-01986-w

**Published:** 2021-05-25

**Authors:** Yitong Liu, Zhizhong Hu, Yang Zhang, Chengkun Wang

**Affiliations:** Hunan Province Key Laboratory of Tumor Cellular & Molecular Pathology, Cancer Research Institute, Hengyang Medical School, University of South China, 28 West Changsheng Road, Hengyang, 421001 Hunan People’s Republic of China

**Keywords:** Epstein–Barr virus, Long non-coding RNAs (lncRNAs), EBV latent infection, EBV lytic infection, Tumourigenesis

## Abstract

Epstein Barr-virus (EBV) is related to several cancers. Long non-coding RNAs (lncRNAs) act by regulating target genes and are involved in tumourigenesis. However, the role of lncRNAs in EBV-associated cancers is rarely reported. Understanding the role and mechanism of lncRNAs in EBV-associated cancers may contribute to diagnosis, prognosis and clinical therapy in the future. EBV encodes not only miRNAs, but also BART lncRNAs during latency and the BHLF1 lncRNA during both the latent and lytic phases. These lncRNAs can be targeted regulate inflammation, invasion, and migration and thus tumourigenesis. The products of EBV also directly and indirectly regulate host lncRNAs, including LINC00312, NORAD CYTOR, SHNG8, SHNG5, MINCR, lncRNA-BC200, LINC00672, MALATI1, LINC00982, LINC02067, IGFBP7‐AS1, LOC100505716, LOC100128494, NAG7 and RP4-794H19.1, to facilitate tumourigenesis using different mechanisms. Additionally, lncRNAs have been previously validated to interact with microRNAs (miRNAs), and lncRNAs and miRNAs mutually suppress each other. The EBV-miR-BART6-3p/LOC553103/STMN1 axis inhibits EBV-associated tumour cell proliferation. Additionally, *H. pylori*–EBV co-infection promotes inflammatory lesions and results in EMT. HPV–EBV co-infection inhibits the transition from latency to lytic replication. KSHV–EBV co-infection aggravates tumourigenesis in huNSG mice. COVID-19–EBV co-infection may activate the immune system to destroy a tumour, although this situation is rare and the mechanism requires further confirmation. Hopefully, this information will shed some light on tumour therapy strategies tumourigenesis. Additionally, this strategy benefits for infected patients by preventing latency to lytic replication. Understanding the role and expression of lnRNAs in these two phases of EBV is critical to control the transition from latency to the lytic replication phase. This review presents differential expressed lncRNAs in EBV-associated cancers and provides resources to aid in developing superior strategies for clinical therapy.

## Introduction

**Fig. 1:**
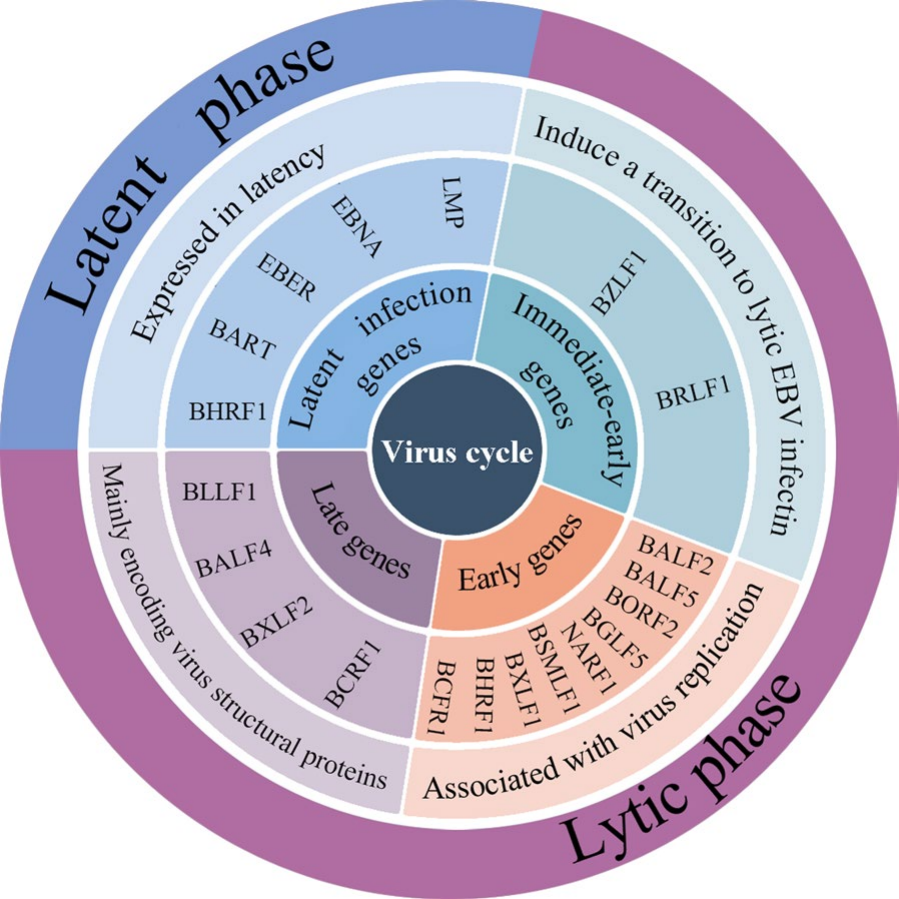
Simplified summary of EBV-encoded genes during latent and lytic phases. Latent infection genes are encoded in the latent phase, such as LMP, EBNA, EBER, and BART. Immediate-early genes, including BZLF1 and BRLF1, encode proteins that promote the transition to EBV lytic infection. Early genes are associated with virus replication and include BALF2, BALF5, BORF2, BGLF5, NARF1, BSMLF1, BXLF1, BHRF1, and BCFR1. Late genes, including BLLF1, BALF4, BXLF2, and BCRF1, mainly encode virus structural proteins. Immediate-early genes, early genes, and late genes are expressed during the lytic phase

Epstein Barr virus (EBV), a gamma-herpesvirus, was first identified in a case of Burkitt lymphoma in 1964 in Africa [1]. Although patients are usually asymptomatic, EBV causes lifelong persistent infection in more than 90% of adults worldwide [2]. Once an infection occurs, it is never cleared, and EBV remains in a subset of B lymphocytes throughout the life of the host [3]. EBV is an oncovirus associated with lymphomas and epithelial cell cancers [4–6] and has two modes of replication: replication via infected B cell proliferation and replication via lytic virion production [7]. Both modes play essential roles in facilitating tumourigenesis. Three distinct processes exist in latency: viral persistence; limited virus expression that alters cell growth and proliferation; and retained potential for reactivation to lytic replication [8]. Typically, the lytic replication phase destroys host cells and is more likely to lead to Hodgkin’s lymphoma, and inhibition of the lytic replication phase may benefit affected patients [7]. According to when they are produced within the virus circle, virus genes can be divided into latent infection genes, immediate-early genes, early genes and late genes (Fig. 1). Latent infection genes include six EBV-encoded nuclear antigens (EBNA-1, -2, -LP [leader protein], -3A, -3B, and -3C), two latent membrane proteins (LMP-1 and -2), two small noncoding RNAs (EBER-1 and -2), Bam-HI A rightward transcripts (BARTs) and Bam-HI H leftward reading frame (BHLF), which are produced during latent infection to stimulate host cell proliferation [7, 9]. Among them, EBNA-1 is necessary for episome replication and virus persistence, and its tyrosine 518 (Y518) forms DNA protein crosslinks and facilitates replication termination at the EBV origin of plasmid replication (OriP). EBNA-2 is required for B lymphocyte immortalization and upregulates the expression of LMP-1 and LMP-2. LMP-1 is also essential for B lymphocyte immortalization and induces multiple activation markers. Additionally, LMP-1 can prevent EBV-infected B cells from undergoing apoptosis [8]. Immediate-early genes are the first group of genes expressed after activation of the virus from the latent state and include BZLF1 and BRLF1, which are transcription factors necessary to transition EBV into the lytic phase of infection. The early genes include BALF5, BALF2, BORF2, NARF1, BGLF5, BXLF1, BHRF1, BMRF1 and BSMLF1, which are associated with virus replication. In addition, the late genes, mainly genes encoding viral structural proteins, are expressed after DNA replication, including BLLF1, BALF4, BXLF2 and BCRF1. Although the genome of EBV and its products have been studied in detail, the transition from latency to the lytic replication phase is remains unclear and the exact mechanism of the genes mentioned above in proliferation, immune escape, invasion and metastasis are not clearly defined tumourigenesis.

Long noncoding RNAs (lncRNAs), which are more than 200 nucleotides in length, were previously believed to be irrelevant in the genome and to not be transcribed [10]. Currently, lncRNAs are typically classified into four types: intergenic, intronic/intragenic, antisense and overlapping [11]. After genomic DNA transcripts to lncRNAs, some of them function in the nucleus, while others are transported to the cytoplasm, where lncRNAs can regulate mRNA stability, and translation and interfere with post-translational modifications in various manner [12]. LncRNA, as competitive endogenous RNA (ceRNA), compete with miRNA to stable mRNA and form double-stranded RNA (dsRNA) with mRNAs to stabilize mRNAs. However, LncRNAs can also act as RNA binding proteins (RBPs) involved in the degradation of mRNA [13]. Recently, lncRNAs were shown to play a regulatory role in gene expression and impact tumourigenesis [14–16]. What’s more, lncRNAs have modest sequence conservation and are highly tissue-specific [17, 18], foreshadowing future diagnosis, prognosis and clinical therapy.

Previous studied have focused on miRNA in regulation of EBV and host cells, however, the wide distribution of lncRNAs in subcellular indicated that lncRNA also played an essential role in various regulatory processes. However, it remains to be further understood which lncRNAs are differentially expressed in the latency and lytic replication phases, and what their exact mechanism and roles are in EBV-infected cells. Herein, we summarize the role of lncRNAs in EBV-associated cancer from the aspects of EBV-encoded lnRNAs during latent phase and lytic phase, the associations between lncRNAs and miRNAs in EBV infection, and pathogens co-infected with EBV. We hope to provide hints concerning the subsequent discovery of biomarkers and clinical diagnosis and clinical therapy.

## EBV-encoded lncRNAs in the latent phase

### BART lncRNAs

In addition to the well-established EBNA, LMP, and EBER genes, EBV has been found to express several spliced RNA transcripts, including BARTs [19]. EBV BARTs consist of two groups non-coding RNAs. One group is microRNAs (miRNAs), which are produced from introns before splicing. The other group is a complex family of alternatively spliced polyadenylated RNAs. These groups contribute to the reprogramming of host cell gene expression [20, 21]. Marquitz et al. believed that the latter group contained functional lncRNAs and suggested that the abundant expression of these lncRNAs facilitates tumourigenesis without the expression of immunogenic proteins. Additionally, previous studies have indicated that some BARTs are mainly found in the nucleus of nasopharyngeal carcinoma (NPC) C666-1 cells and that some BARTs are not exported to the cytoplasm [22, 23]. Similarly, Verhoeven et al. hypothesized that BART lncRNAs reside in the nucleus of EBV-infected cells [24]. In addition, the most significant characteristic of EBV latency in NPC is high expression of BARTs, including lncRNAs and miRNAs, as there are few other EBV-derived products present during the latent phase [25]. It is reasonable that the expression of BARTs may be related to the oncogenic state in NPC.

Verhoeven et al. indicated that BART lncRNA-induced dysregulation of the expression of genes may result in promotion of cancer progression and metastasis and evasion of the immune response in NPC [24]. Aiolos, encoded by the IKZF3 gene, a lymphoid-restricted transcription factor, interacts with lkaros to modulate lymphocyte differentiation and usually not expressed in epithelial cells [26]; however, Aiolos is only expressed in EBV-positive NPC C666-1, the expression of IKZF3 is positively correlated with that of BART lncRNA isoform RPMS1, a size of approximately 4 kb and the most abundant BART transcript species in NPC [24]. In addition, inhibition of BART lncRNAs by GapmeRs results in downregulation of IKZF3 expression, followed by upregulation of p66Shc mRNA expression, but the protein level does not increase [24]. Several studies have demonstrated that the Src homology/collagen (Shc) adaptor protein plays a key role in apoptosis and the cellular redox state [27, 28]. In NPC C666-1 cells, overexpression of BART lncRNAs was found to be negatively correlated with the expression of IL-6 by induced by GapmeRs. Similarly, several interferon genes, including IFNL1, IFNL2, IFNA1 and IFNB1, were apparently upregulated, and the cytokine genes IL5, IL10 and CXCL8, various interferon-stimulated genes (ISGs; ISG20, OAS2, IFIT1 and IFIT2), and CXCR2, which is a chemokine-related gene, were also upregulated by knockdown of BART lncRNAs in C666-1 cells, most of the genes above are related to the inflammatory and immune response, suggesting that EBV BART lncRNAs might regulate a host immune response that promotes immune evasion. Notably, IFIH1, which encodes MDA5, a cytoplasmic sensor of viral nucleic acids in host antiviral responses, is negatively correlated with BART lncRNAs. Additionally, Verhoeven also confirmed that BART lncRNA significantly inhibits mitochondrial antiviral signalling (MAVS)-induced IFN-β promoter activity [24]. These results further confirm that BART lncRNAs play an immunomodulatory role. However, how do BART lncRNAs regulate the host genes associated with innate immunity? Verhoeven et al. demonstrated that BART lncRNAs block the expression of Pol II to regulate IFNB1 and CXCL8 expression and modulate host gene expression through interaction with CREB-binding protein (CBP) in EBV-infected cells by using indirect immunofluorescence and RNA FISH. Additionally, the expression of BART lncRNAs inhibited MAVS protein-induced histone acetyltransferase (HAT) activity and maintained latent EBV infection by influencing histone acetylation and methylation processes. These findings further suggest that during the gene transcription process, BART lncRNAs are also key regulators of chromatin remodelling. Besides, EBV BART lncRNAs upregulate the well-established biomarker Septin 9, which is hypermethylated in several cancers.

Likewise, Marquitz et al., using qRT-PCR, confirmed that BART lncRNAs downregulate several genes, including the tumour proapoptotic gene RNF144B; the secreted gastric protease-encoding gene PGC; the angiogenic factor-encoding gene VEGFA; ATF5 and SLC7A11, which are genes involved in cell adhesion and the unfolded protein response; and the migration-related genes RASIP1, 441 VEGFA, CDH11 and ITGA6, in six AGS-EBV cell lines [29]. Similarly, compared with an EBV-negative NPC cell line, the C666-1 cell line (which has high levels of BARTs) showed decreased expression of the ITGA6, CDN11, SLC7A11 and RASIP1 genes [29]. These results further suggest that the above genes share common functional mechanisms related to BART lncRNAs. The most widely recognized strategy by which lncRNAs silence genes is via the recruitment of Polycomb repressive complex 2 (PRC2) to remove the H3K27me3 marker [30]. Additionally, a prominent feature of EBV-positive gastric cancer (GC) is very high levels of CpG island promoter methylation, leading to the silencing of many important genes [31–33]. Marquitz et al. concluded that BART lncRNAs might contribute to the DNA methylation phenotype or influence histone regulation [29].

**Fig. 2:**
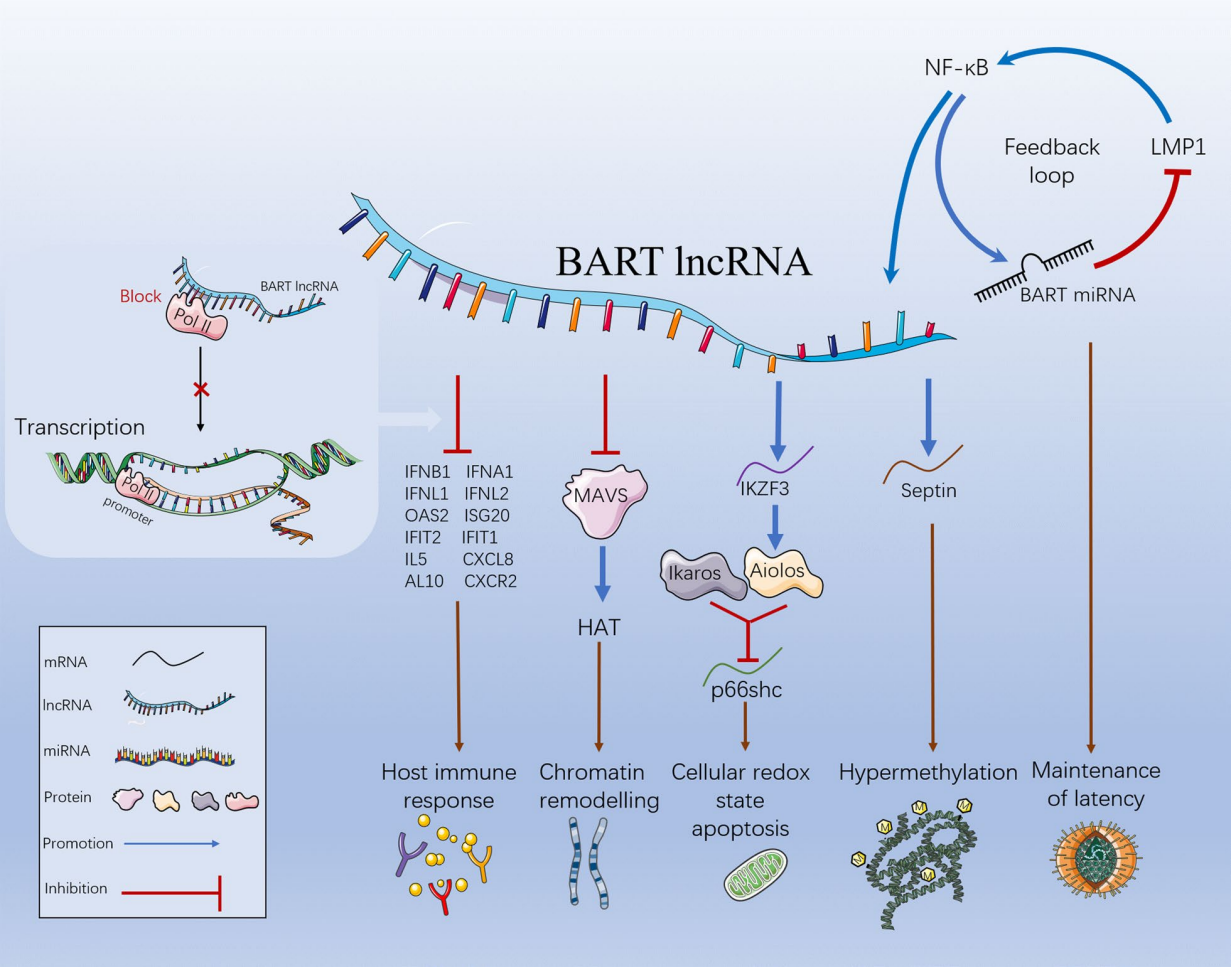
Role of BART lncRNAs encoded by EBV. BART lncRNAs inhibit the host immune response by blocking Pol II, and the genes involved in the immune response cannot be transcribed. BART lncRNAs inhibit MAVS to contribute to chromatin remodelling. BART lncRNAs promote the activation of IKZF3 to regulate the cellular redox state and apoptosis. BART lncRNAs induce hypermethylation via Septin. The feedback loop among BARTs, NF-κB, and LMP1 facilitates the persistence of latency

Verhoeven et al. further confirmed a feedback loop among LMP1, BARTs, and NF-κB in NPC and showed that activated NF-κB signals promoted EBV latent infection. The expression of BART lncRNAs and BART miRNAs was upregulated by LMP1 activated BART promoters through NF-κB signalling, and the p50 and p65 NF-κB subunits could bind to the BART promoters; however, high expression of BART miRNA (mainly miR-BART5-5p, and to a lesser extent by miR-BART3-3p and miR-BART7-3p, but not miR-BART13-3p or miR-BART14-3p) downregulated the expression of LMP1, providing negative feedback [25]. Inhibition of NF-κB signalling actually leads to BART overexpression and lytic replication [25]. It is reasonable to speculate that this feedback loop plays a role in maintaining persistent latent infection and avoiding entry into the lytic phase. (Fig. 2).

### BHLF1 lncRNAs

Previous studies have indicated that the BHLF1 gene encodes several circular and a large number of linear RNAs that play non-coding roles during virus replication [34, 35]. However, Yetming et al. recently demonstrated that BHLF1 is also transcribed during latency and may function mainly instead via its transcript as a lncRNA to contribute to viral latency and B cell immortalization [36]. However, the specific role of BHLF1 lncRNA in tumourigenesis is still not clear. In fact, BHLF1 is known as an early lytic cycle gene [37]. Park et al. showed that the GC-rich EBV BHLF1 lncRNA was always present in virus-induced nodular structures (VINORCs) using FISH, which mediate the processing of pre-mRNA and the output of mRNA during lytic infection and thus lead to viral host shutoff (VHS). BHLF1 lncRNA may not play a role in VINORC assembly, however, a series of twelve 125 bp tandem repeat sequences within BHLF1 suggests that lncRNA may play scaffolding and structural roles in recruitment of RNA-binding proteins and splicing factors to VINORCs. Seven proteins (SC35, SON BMLF, Y14, SRp20, NXF1, ALY) are recruited to VINORCs and play roles in pre-mRNA splicing and mRNA nuclear export. However, the exact mechanism by which BHLF1 lncRNA recruits the above proteins requires further research [37].

## EBV-encoded lncRNAs in the lytic phase

Latent infection is critical to the long-term survival of the host; in the latent phase, the virus does not replicate autonomously in the host, and its genome is replicated as the host cell replicates. Spreading of the virus from one cell to another and from one host to another requires lytic replication [38]. EBV lncRNAs might also act as key regulators of viral lytic replication and activate the expression of early and late genes by altering EBV chromatin structure [39]. Dresang et al. and O'Grady et al. indicated that more than a hundred novel lytic virus long non-coding RNAs are expressed by EBV [40, 41]. Cao et al. indicated that the origin of replication (oriP) in EBV latent infection is transcribed bidirectionally at the time of reactivation, with the leftward oriP (oriPtL) and the rightward oriP (oriPtR) mostly located in the nucleus, indicating that oriPtL-derived vlncRNAs promote the expression of lytic viral genes and contribute to the viral replication cascade. The transcripts of the oriPtL and oriPtR are viral late genes [38]. NONO is a multifunctional paraspeckle DNA binding protein that plays a role in transcriptional regulation [42, 43]. Cao et al. found that NONO binds to oriP transcripts in the interaction between oriPts and the antiviral/stress response pathway, suggesting that these vlncRNAs are associated with paraspeckle-based innate antiviral immune pathways and contribute to the viral lytic cascade [38].

Similarly, O'Grady found that EBV lncRNAs binds to the viral genome in a heteroduplex manner and tags DNA for packing and concentrating for nuclear export of the newly synthesized viral genome [40]. Previous studies have mentioned that BHLF1 is found in VINORCs [37]. It is therefore reasonable to speculate that BHLF1 facilitates virus replication and spread.

Furthermore, lytic lncRNAs are potentially packaged in virions and might carry essential messages to promote the initial infection of epithelial cells or naïve B cells [38]. Gallo et al. found 9 lncRNAs (SNHG5, H19 antisense, H19 upstream conserved 1&2, H19, 7SL, HOXA6as, NDM29, Tsix and HAR1B) in the cargo of the lymphoblastoid cell line (LCL) exosome cargo [44]. H19, one of the major cancer genes, is highly expressed in almost all cancers and participates in all phases of tumourigenesis [45, 46]. 7SL expression is obviously upregulated in EBV-infected cells and repressed p53 translation in cancer cells. Moreover, the promoter sequences of 7SL resemble EBER genes [47–49]. These results indicated that lncRNAs play a leading role in regulating of the tumour microenvironment. Besides, exosomes promote tumour immune escape and tumourigenesis [50, 51]. However, the mechanisms of exosome-related lncRNAs in immune escape is remain unclear. Hence, further exploration of the role of lncRNAs in exosomes in immune escape would be meaningful, and may provide new sights for clinical therapy.

## Host lncRNAs regulated by EBV

**Fig. 3:**
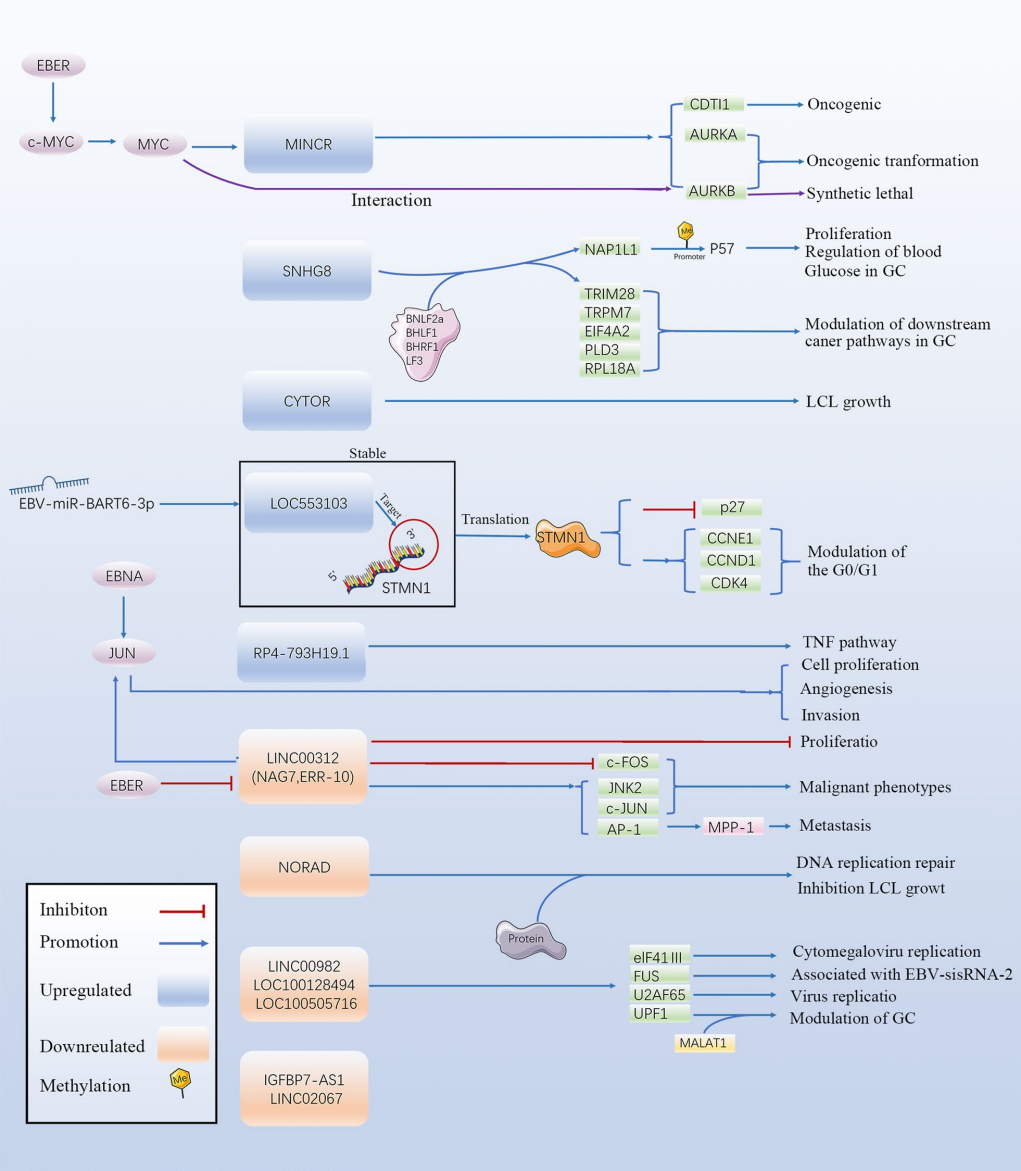
Host lncRNAs involved in EBV-associated cancer. The lncRNAs MINCR, SNHG8, CYTOR, LOC554103, and RP4-793H19.1 are upregulated, and the lncRNAs LIN00312 (also known as NAG7 and ERR-10), NORAD, LINCOO982, LOC10028494, LOC100505716, IGFBP7-AS1, and LINCO2067 are downregulated in EBV-associated cancer. Both upregulated and downregulated lncRNAs are involved in tumourigenesis

**Fig. 4:**
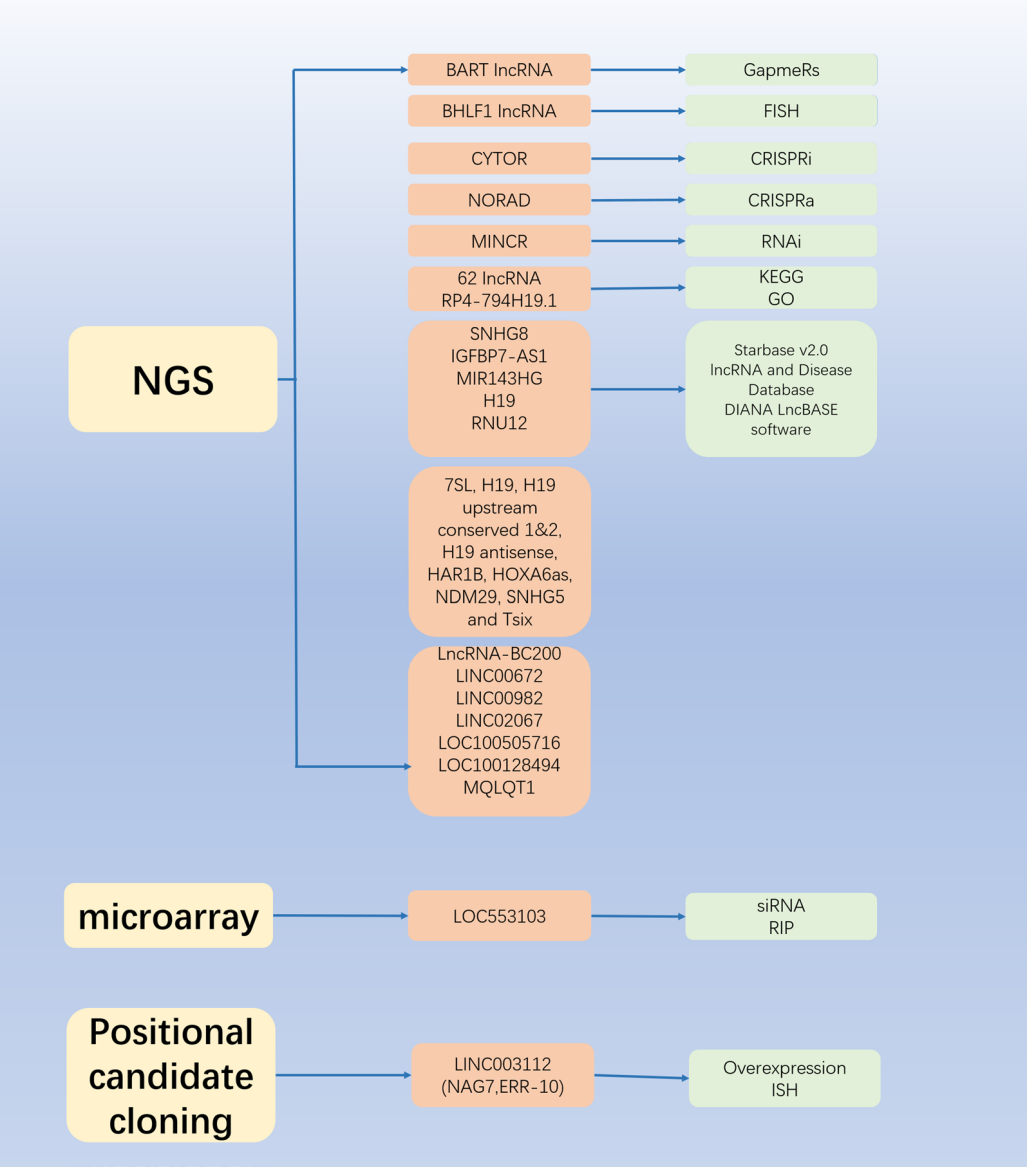
Flowchart of the research methodology for each study. The differential expression of most lncRNAs was identified by NGS. LOC553103 was identified by microarray. LINC003112 was identified by positional candidate cloning. BART lncRNA was knocked down using the Gapmers technique for further study. BHLF1 lncRNA was identified by FISH. CYTOR was knocked down by CRISPRi, and NORAD was overexpressed by CRISPRa. MINCR was further evaluated using RNAi. Sixty-two lncRNAs and RP4-794H19.1 were predicted to be involved in their target pathways by KEGG and GO analyses. SNHG8, IGFB7-AS1, MIR143HG, H19, and RNU12 were predicted to be target genes using starBase v2.0, lncRNA and Disease Database and DIANA LncBASE software. LOC553103 was further assessed using siRNA and RIP. LINC003112 was further studied using an overexpression plasmid and was identified in tissue samples for analysis relevant to ISH. *NGS* next-generation sequencing, *FISH* fluorescence in situ hybridization, *RNAi* RNA interference, *CRISPRi* CRISPR interference, *CRISPRa* CRISPR activation, *RIP* RNA-binding protein immunoprecipitation, *ISH* in situ hybridization

Not only does the lncRNA encoded by EBV have an impact on the host, but EBV also regulates the expression of host lncRNAs, and this regulation has been confirmed to play both positive and negative roles in proliferation, invasion, immune escape and cell cycle (Figs. 3, 4).

Zhang et al. found that the expression of LINC00312, also termed as NAG7 and ERR-10 (represented by NAG7) [52], is downregulated in NPC, and NAG7 expression is positively associated with lymph node metastases and negatively related to clinical stage and tumour size. Moreover, the expression of NAG7 is indirectly downregulated by EBER-1, but the mechanism requires further study [53]. NAG7 can distinguish between non-cancerous and NPC patients, thus it may be a biomarker to diagnose and predict the clinical outcome of NPC.

NAG7 facilitates cell invasion by activating JUN pathways in NPC [54]. EBNA1 regulates the JUN transcription factor pathway in NPC and promotes angiogenesis in vitro [55]. Furthermore, the expression of JUN family members promotes cell proliferation in diffuse large B cell lymphomas [56]. The MAPK pathway, comprising c-Jun NH2-terminal kinase (JNK), ERK, and p38, is generally activated in human cancers, resulting in malignant phenotypes and is associated with metastatic behaviour in many cancer types [57–59]. NAG7 upregulates the expression of JNK2 and c-Jun and downregulates c-Fos, but ERK and p38 are not affected, leading to enhanced transcriptional activation of AP-1, followed by the transcription of MMP-1 [54]. MMP-1 may also affect the cytoskeletal arrangements considered to associate with different cell adhesion molecules (such as CD44 and integrins) to promote cell adhesion and motility [60]. Huang et al. shown that NAG7 overexpression increases the adhesion, invasion and motility of NPC HNE1 cells in vivo and in vitro via JNK2/AP-1/MMP1 [54]. Additionally, the Ras/Raf pathway is the upstream regulator that activates the MAPK signalling pathway in human cancers. Previous studies also suggested that NAG7 activates the H-Ras/p-c-Raf pathway to promote tumourigeneses [54]. However, NAG7 prevents the cell cycle transition from G1 to S phase, inhibiting cell proliferation and inducing apoptosis in NPC [61–63]. Although the exact mechanism requires further study, NAG7 plays dual roles in the proliferation and invasion of NPC cells.

Additionally, Wang et al. identified lncRNAs in LCLs, including NORAD and CYTOR, which were differentially expressed during EBV infection by RNA-seq [64]. NORAD can bind to proteins that participate in DNA replication and repair [65]. What’s more, the proteins PUMALIO1 and PUMILIO 2 bind to the PUMILIO response element (PRE) of the 8-nt sequence at the 3ʹUTR of target mRNAs, subsequently activating mRNA deadenylation and decapping, and leading to accelerated turnover and decreased translation [66]. However, studies have shown that NORAD prefers to bind to PUM1/2 in the human cell cytoplasm because it contains 15–17 PREs and prevents the target mRNAs from degradation [67, 68]. Lee et al. found that PUMILIO drives chromosomal instability by hyperactively repressing mitotic, DNA repair, and DNA replication factors in the absence of NORAD [69]. CYTOR is essential for breast cancer cell cytoskeleton maintenance, proliferation, and migration and is upregulated in multiple cancers [70, 71]. However, Wang et al. knocked down CYTOR with CRISPRi and upregulated NORAD with CRISPRa to significantly reduce LCL growth, suggesting that regulation of CYTOR and NORAD by EBV contributes to LCL survival and growth [64]. However, the exact mechanism between EBV and host lncRNAs needs to further study. In addition, further characterization of the role of the above lncRNAs would be interesting.

Huang et al. used deep sequencing to identify five EBVaGC-specific lncRNAs, RNU12, H19, SNHG8 and RP11-359D14.3, further identified the expression level by RT-PCR, H19, RNU12 and MIR143HG were identified as non-significant while RP11-359D143.3 was removed because of its unsatisfactory quality control results. Notably, lncRNA SNHG8 affected several GC-specific pathways by interacting with the EBV genes BHRF1, LF3, BHLF1 and BNLF2a and regulated the expression of TRPM7, TRIM28, EIF4A2, RPL18A, PLD3, and NAP1L1 [72]. The EBV genes BHLF1 and leftward reading frame 3 (LF3) were found in the polyribosome fraction of EBV-infected cells and were transcriptionally expressed in virus-associated tumours [73], both are associated with the lytic replication cycle, mainly in epithelial cells [74]. Furthermore, BHRF1 has 38% sequence homology with the antiapoptotic protein Bcl-2 and the function of BHLF is also similar to that of Bcl-2, leading to persistent virus infection and contributing to tumourigenesis [75]. Additionally, BNLF2a, an early gene, may contribute to immune escape because its protein has two domains(a hydrophilic, cytosolic N-terminal domain and a hydrophobic, membrane-spanning C-terminal domain) both of which are associated with the disruption of viral peptide transport into the endoplasmic reticulum and of peptide loading onto human leukocyte antigen class I molecules, leading to the reduction of endogenous antigen presentation and preventing recognition by CD8+ T-cells [76, 77]. TRIM28, TRPM7 and NAP1L1 influence downstream cancer pathways in GC. TRIM28 contributes to tumourigenesis and acts as an independent prognostic factor for poor survival in GC [78]. NAP1L1 regulates the methylation of the P57 (Kip2) promoter to influence the proliferation of pancreatic neuroendocrine tumour cells [79]. Moreover, Chen et al. proved that higher SNHG8 expression was associated with a later TNM stage in GC. Although no significant correlation was found between SNHG8 expression and tumour size, higher expression of SNHG8 was found in larger tumours, further demonstrating that SNGH8 promotes the development of tumours as a proto-oncogene [80]. The lncRNA SNHG5 is downregulated and correlated with TNM stage in GC [81]. Lin et al. confirmed that elevated fasting blood glucose levels and high levels of SNHG8 expression after radical gastrectomy predict a poor prognosis [82]. However, a recent study suggested that the lncRNA SNHG8 suppresses cell growth in EBV-associated GC [83]. These results reveal the regulation between lncRNAs and EBV in GC and provide a comprehensive understanding of the mechanism of immune escape, although the exact mechanism warrants further study. Additionally, the EBV-specific lncRNAs suggest that they may serve as predictive biomarkers.

MYC, a transcription factor, was first identified in Burkitt lymphoma (BL) but is expressed in all lymphomas (not only in BL) and affects the expression of several genes, leading to the development of many neoplasms [84–86]. Doose et al. identified a MYC-regulated lncRNA named MYC-induced long noncoding RNA (MINCR) that affects the expression of MYC-regulated cell cycle genes to control cell cycle processes. The expression levels of AURKA, AURKB and CDT1 are significantly affected by MINCR [87]. Furthermore, AURKA and AURKB have been shown to play essential roles in MYC-induced oncogenic transformation, and AURKB interacts with MYC, showing synthetic lethality [88]. A previous report suggested that CDT1 plays an oncogenic role in the progression of lymphoma [89]. What’s more, Marquitz et al. also demonstrated that MYC was activated during EBV latent infection in AGS cells [29]. In type III latency, c-MYC is directly activated by EBNA2 [90]. However, as a modulator of the MYC transcriptional program, the mechanism by which MINCR regulates cell cycle progression in EBV-associated cancers must be further explored.

Zhang et al. identified eight lncRNAs in four EBV‐positive cells with RNA-seq and qPCR analyses. LncRNA‐BC200, LINC00672 and MALAT1 were significantly upregulated, while LINC00982, LINC02067, IGFBP7‐AS1, LOC100505716, and LOC100128494 were expressed at low levels. In AGS cells (EBV-positive GC cells), lncRNA BC200, LINC00672, MALAT1 and LOC100128494 were significantly upregulated, while LINC02067 was downregulated. In C666-1 (EBV-positive NPC cells), lncRNA BC200, LINC00672 and LOC100128494 were upregulated while MALAT1 and LOC100505716 were downregulated. In addition, Zhang et al. demonstrated that the six lncRNAs other than IGFBP7‐AS1 and LINC02067 (for which no data were available) target several common genes, including eIF4AIII, UPF1, FUS, and U2AF65, using egalovirus replication [91, 92] These results indicate that EBV may dysregulate host lncRNAs to regulate its own replication. Recent studies have reported that UPF1 regulates the development of GC by affecting the lncRNA MALAT1 [93]. Tompkins et al. predicted that FUS is associated with ebv stable intronic sequences (sisRNA2), a class of ncRNAs [94]. Moreover, Domsic et al. suggested that the general splicing factor U2AF65 modulates virus replication [95]. Furthermore, Kara et al. hypothesized that the lncRNA M3-04 is regulated by TMER8-derived antisense miRNAs and is relevant to reinforced lytic replication in vivo based on experiments in a murine model [96]. The above lncRNAs profile provides a resource to explore the mechanism of tumourigenesis between EBV and host lncRNAs.

Li et al. based on next-generation sequencing (NGS) analysis, suggested that 62 lncRNAs trans-regulated genes participate in the EBV infection pathway and that the proto-oncogene JUN was related to the cis-regulatory lncRNA RP4-794H19.1 and contributes to the tumour necrosis factor (TNF) signalling pathway in NPC, although these ideas need further practical validation [97]. Additionally, NGS, a high-throughput technique based on massive, small reads of the genome, enables rapid sequencing of the base pairs in DNA or RNA samples and the genomics data come from Whole Exome Sequencing (WES) and RNA sequencing (RNAseq). Kosvyra et al. suggested that utilizing the above data to create an integrated profile of a patient will allow better understanding of the disease and using chronic lymphocytic leukaemia (CLL) as an example to show that the profile efficiently summarizes the large-scale datasets including the results with the clinical profile and indicators from different data types [98]. Using NGS in patients with EBV-positive NPC, the previously described polymorphism in the promoter of the lytic transactivator of BZLF1 is associated with increased lytic replication [99, 100]. The profile based on NGS facilitates medical research seeking differentially expressed genes and provides new possibilities for prognostic and precision medicine. These results suggest that products encoded by EBV profoundly affect host lncRNA regulation representing a potential biomarker for invasion, tumourigenesis and prognosis. Additionally, lncRNAs may be a potential target for clinical therapy and the treatment strategy must be further explored.

Agsalda-Garcia et al. proposed that a Raman-enhanced spectroscopy (RESpect) probe, which enhances Raman spectroscopy technology using portable fibreoptic devices, may identify potential markers for diagnosis by identifying the molecular chemical composition of tissues and cells [101]. Raman spectroscopy (RS) identifies chemical and molecular fingerprints of materials using inelastic scattering of photons with molecular bond vibrations, resulting in frequency energy shifts [102], therefore, RS has potential to identify differentiated tumour tissue. More importantly, the advantage of RESpect is its rapid, real-time assessment and non-invasiveness [103]. Agsalda-Garcia et al. discriminated childhood non-Hodgkin lymphomar (NHL) subtypes by standard RS instrumentation and the RESpect probe, confirming the feasibility of the RESpect probe [101]. RESpect, as a potential real-time screening instrument, can change the paradigm of screening for cancers. Additionally, RESpect provides the resource for researchers to explore novel molecules in tumours that may serve as biomarker for diagnosis or play a key role in regulating in tumourigensis.

## Associations between lncRNAs and RNAs in EBV infection

LncRNAs have confirmed that interacting with RNAs (mRNAs or miRNAs) in various way plays a regulatory role in tumourigenesis [13]. LncRNA forms double-stranded RNA (dsRNA) with mRNA to regulate mRNA stability. Faghihi et al. found that beta-secretase-1 (BACE1) mRNA combines with BACE-1 antisense transcript (BACE1-AS) to contribute to BACE1 mRNA stability, leading to increased BACE1 protein expression in Alzheimer’s disease [104]. By contrast, Gong et al. suggested that lncRNA half-STAU1-binding site RNA(1/2-sbsRNAs) forms dsRNA with mRNA and recruit STAU1 to bind to mRNA and degrade mRNA [105]. What’s more, lncRNAs can compete with endogenous RNAs and inhibit each other, thus establishing a regulatory ceRNA network (lncRNAs–miRNAs–mRNAs) that modulates target mRNAs [106]. There is increasing evidence supporting the existence of interactions between lncRNAs and miRNAs or mRNAs in several cancers [107–109] while EBV-associated cancers are no exception.

An aberrant lncRNA-mRNA-miRNA ceRNA network is also present in EBV-associated cancer. He et al. hypothesized that EBV-miR-BART-6-3p downregulates lncRNA LOC553103 to inhibit epithelial–mesenchymal transition (EMT), cell invasion and migration in NPC [110]. Additionally, Wang et al. indicated that the EBV-miR-BART6-3p/LOC553103/STMN1 axis inhibits EBV-associated tumour cell proliferation by modulating the expression of cell cycle-associated proteins. LOC553103 can directly target the 3′UTR of STMN1 mRNA, thereby stabilizing its structure and facilitating STMN1 translation. Knockdown of L0C553103 or STMIN1 can upregulate p27 and decrease CCNE1, CCND1, and CDK4, which are proteins involved in the G0/G1 cell cycle checkpoint [111]. These findings provide new insights into novel targeted therapies.

In addition, using miRwalk datasets, Jing et al. identified that both CXCL10 and GDF5 were in the ceRNA network and that they can be regulated by miR-6877-3p and two previously unreported lncRNAs (TP73-AS1 and RP5-1039K5.19). Eck et al. indicated that CXCL10, a strong angiostatic factor, is related to tumour-infiltrating T cells in GC [112]. GDF5, a growth differentiation factor, has proved that participates in EMT in salivary gland pleomorphic adenoma [113] and regulates TGFβ-dependent angiogenesis in breast carcinoma [114]. Additionally, TP73-AS1 and RP5-1039K5.19 were identified in the ceRNA regulation network and have been identified as candidate targets in in-depth study on EBVaGC [115]. Although the mechanism mentioned above remains unclear, recognizing the crosstalk between viral miRNAs and lncRNAs is beneficial and will provide a theoretical and practical basis to determine the effect of EBV infection on carcinogenesis.

Sethuraman et al. provided bioinformatics evidence that Kaposi’s sarcoma herpesvirus (KSHV) and EBV miRNAs interact with cellular lncRNAs in an RNA-induced silencing complex (RISC)-dependent manner and demonstrated that lncRNAs participate in non-canonical miRNA binding, in which they bind to the 3ʹ end of both viral and cellular miRNAs, more frequently than mRNAs. What’s more, Sethuraman et al. suggested that miRNA–lncRNA interactions occur in both the nucleus and cytoplasm, although the mechanism must be further proven experimentally [106]. Furthermore, lncRNAs can bind to miRNAs, whether they are cellular or viral miRNAs, inhibiting miRNA-targeted mRNAs [116]. This information provides a resource to explore the biological correlation between lncRNAs and RNAs, and provides additional evidence that EBV-encoded genes regulate host lncRNAs.

## EBV co-infectious agents

**Fig. 5:**
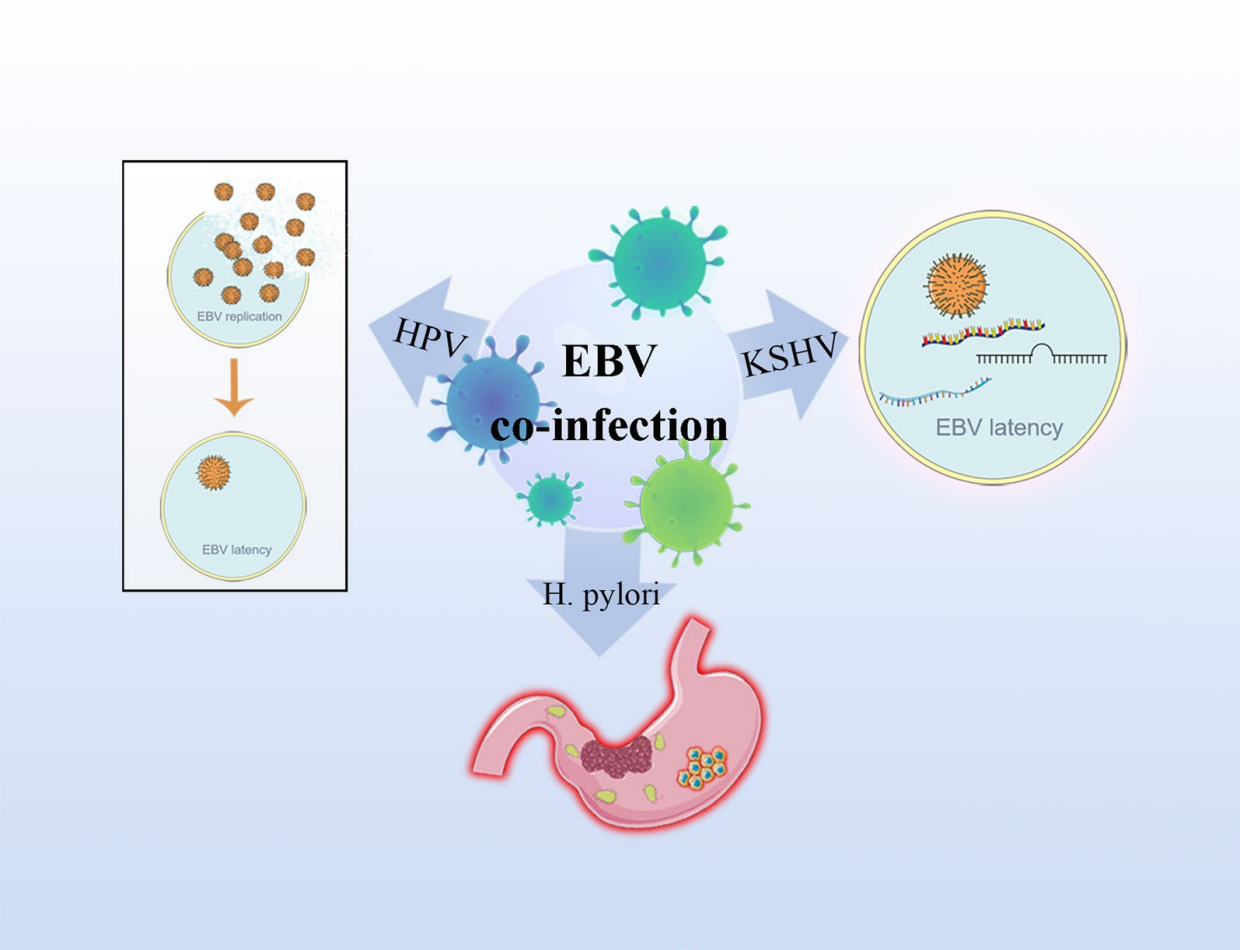
EBV co-infectious agents. *H. pylori*-EBV co-infection not only enhances the inflammatory process but also promotes immune evasion, EMT and bacterial growth and inhibits apoptosis. HPV–EBV co-infection inhibits EBV lytic replication and shifts EBV towards the latent phase. KSHV–EBV co-infection aggravates tumourigenesis by mutually reinforcing the persistence of each latent genome and altering cell proliferation

Viral co-infection has received increased attention, and identifying new mechanisms may provide new insight into novel clinical therapies (Fig. 5).

*Helicobacter pylori* (*H. pylori*) is currently recognized as a carcinogen and has been found to be related to several diseases of the stomach [117, 118]. Carrasco et al. suggested that co-infection exacerbates inflammatory lesions compared with infection with only *H. pylori* or EBV [119]. In addition, Cardenas-Mondragon et al. indicated that GC with co-infection with EBV and *H. pylori* was related to premalignant lesions and intestinal-type GC [120]. The two pathogens have developed mechanisms that restrain the host inflammatory response and promote chronic colonization of the stomach, and infections in this area subsequently develop into chronic gastritis via induction of inflammation and lead to carcinogenic modifications. Yang et al. pointed out that CagA-positive *H. pylori* strains promote cell proliferation and migration by inducing the expression of NF-κB, which binds to the promoter of miR-223 to target ARID1A [121]. Previous studies have reported that NF-κB also upregulates BARTs, including BART lncRNAs and BART miRNAs [25]. The relationship between these factors needs to be further explored. In addition, Liu et al. found that 23 lncRNAs were upregulated and 21 were downregulated in GES-1 cells infected with *H. pylori.* In particular, the lncRNAs XLOC_000620, XLOC_005912 and XLOC_004562 were apparently upregulated; however, XLOC_014388 and XLOC_004122 were expressed at low levels [122]. Furthermore, the let-7 and miR-200 families were downregulated by EBV and *H. pylori* co-infection, which led to enhanced inflammation and resulted in EMT. EBV and *H. pylori* also upregulate miR-155, thereby inhibiting the release of proinflammatory cytokines. *H. pylori* infection causes aberrant expression of lncRNAs, while EBV encodes its own viral ncRNAs, including lncRNAs and miRNAs. However, how *H. pylori* and EBV lead to tumourigenesis remains unclear.

Human papillomavirus (HPV) has been found to be related to cancers of the anogenital tract [123]. However, Guidry et al. infected EBV directly into HPV16-immortalized tonsillar cells and concluded that HPV E6 and E7 interfere with EBV early protein EA-D expression, leading to inhibited EBV lytic replication and a possible transition to EBV latent infection [124]. Nawandar et al. indicated that KLF4, which is downstream of E7, activates the expression of the EBV immediate-early genes BZLF1 and BRLF1, promoting the transition from latency to lytic replication [125]. In addition, studies have shown that differentiated cells express KLF4, PRDM1 and cellular transcription factors that activate the expression of immediate-early genes as well as LMP1 to promote EBV replication [125–127].

KSHV has been found to be related to multicentric Castleman’s disease (MCD), which is caused by B cell lymphoproliferative disorders [128]. Additionally, McHugh et al. demonstrated that KSHV increases EBV-associated tumourigenesis by activating of lytic EBV replication in huNSG mice [129]. Latent nuclear antigen (LANA) of KSHV shows effects similar to EBNA1, leading to persistence of episomal KSHV DNA in proliferating cells. Moreover, Bigi et al. highlighted that the complex interaction between KSHV and EBV aggravates the progression of cancer by carefully maintaining each latent genome and modifying cell proliferation [130].

SARS-CoV-2 (COVID-19), a pathogenic single-stranded RNA human coronavirus, continues to spread all globally and seriously affects human health [131]. In general, human CoV would not cause a deadly disease. However, human lack pre-existing natural immunity for zoonotic origin of COVID-19. However, humans lack pre-existing natural immunity due to the zoonotic origin of COVID-19. Additionally, the recent emergence of mutant strains suggests that the virus has adapted in humans to survive. Mutations in non-structural proteins may enhance replication of the viral genome. Furthermore, mutations within accessory proteins contribute to evasion or regulate host innate immunity, causing persistent virus replication. Parvez et al. considered that further rigorous genetic and molecular studies would update treatment and preventive strategies for recently emerged variants [131]. Additionally, Challenor et al. reported a case in which an EBV-positive Hodgkin lymphoma patient was diagnosed with COVID-19 4 months later, palpable lymphadenopathy was reduced, and a PET/CT scan showed widespread resolution of the lymphadenopathy and reduced metabolic uptake throughout [132]. Challenor et al. hypothesized that COVID-19 infection triggered an anti-tumour immune response and assumed that the mechanisms for this included cross-reactivity of pathogen-specific T cells with tumour antigens and the activation of natural killer cells by inflammatory cytokines produced by infection [132]. Although the assumption must be further explored and validated, understanding the mechanism of the above relationship will be valuable to cancer treatment.

## Conclusion

Although most individuals infected with EBV are asymptomatic, EBV can cause various cancers, including lymphoma, NPC and GC. Viral infection is a dynamic balancing process involving host immunity. Most studies have reported the effect of miRNAs on tumourigenesis, but studies of the effect of lncRNAs on tumourigenesis are rare. The presence of these lncRNAs determines their influence on the body and whether they have positive or negative effects; however, the mechanism of their action must be further explored.

Compared with previous studies limited to miRNAs and proteins encoded by EBV, we focused on the introduction of lncRNAs in EBV-infected cells. We showed that EBV produces not only miRNAs but also lncRNAs. Regarding BART transcripts, one group comprised miRNAs, and the other group comprised lncRNAs. BART lncRNAs affect multiple inflammatory cytokines and promote immune escape. BHLF1 lncRNA has been suggested to play an important role in virus replication and spread. Additionally, host lncRNAs are directly or indirectly regulated in EBV-infected cells and are involved in proliferation, invasion, metastasis, and immune escape, such as lncRNA NAG7, CYTOR, NORAD, SNHG8, MINCR, lncRNA-BC200, LINC00672, MALAT1, LOC100128494, lncRNA RP4-794H19.1, LOC553103, TP73-AS1 and RP5-1039K5.19. Furthermore, lncRNAs are characterized by modest sequence conservation, are highly tissue-specific and can be used as good biomarkers for diagnosis, prevention and even monitoring during treatment. We also discussed the lncRNAs–miRNA–mRNA network, indicating that lncRNAs not only directly target mRNAs but also interact with miRNAs to indirectly regulate the expression of mRNAs.

We also discussed EBV co-infectious agents. *H. pylori*–EBV co-infection promotes inflammatory lesions and results in EMT. HPV–EBV co-infection inhibits the transition from latency to lytic replication. KSHV–EBV co-infection aggravates tumourigenesis in huNSG mice. COVID-19–EBV co-infection may activate the immune system to destroy the tumour, although this situation is rare, and the mechanism must be further confirmed. Hopefully, this information will shed some light on tumour therapy strategies.

RESpect detects molecular chemical fingerprints rapidly and non-invasively, providing a method to identify more differential lncRNAs. Additionally, utilizing this approach may play a significant role in diagnosis, screening cancer and monitoring during treatment.

Furthermore, prevent latency into lytic replication in infected patients is considered beneficial. Although most latently infected B cells do not undergo lytic replication, latency can transition to the lytic replication phase. Understanding the role and expression of lncRNAs in these two phases of EBV is critical to control the transition from latency to the lytic replication phase.

In summary, we present differential lncRNAs in EBV-associated cancers, providing resources and novel insights for future diagnosis, prognosis and clinical therapy.

## Data Availability

Not applicable.

## Abbreviations

EBV: Epstein–Barr virus
lncRNA: Long non-coding RNA
EBNA: EBV-encoded nuclear antigens
LP: Leader protein
LMP: Latent membrane protein
EBER: EBV‐encoded RNA
BARTs: Bam-HI A rightward transcripts
BHLF: Bam-HI H leftward reading frame
HGNC: HUGO Gene Nomenclature Committee
CSTs: Complementary strand transcripts
ORFs: Open reading frames
microRNA: MiRNA
Shc: Src homology/collagen
MAVS: Mitochondrial antiviral signalling protein
HAT: Histone acetyltransferase
VINORCs: Virus-induced nuclear structure
Vhs: Viral host shutoff
vlncRNA: Virus long non-coding RNA
oriP: Origin of replication
oriPtLs: Origin of replication with leftward
oriPtRs: Origin of replication with rightward
NPC: Nasopharyngeal carcinoma
GC: Gastric cancer
EBVaGC: Epstein–Barr virus associated gastric cancer
LCL: Lymphocyte line
BL: Burkitt lymphoma
EMT: Epithelial–mesenchymal transition
dsRNA: Double-strain RNA
KSHV: Kaposi’s sarcoma-associated herpesvirus
RISC: RNA-induced silencing complexes
ceRNA: Competing for endogenous RNA
RESpect: Raman-enhanced spectroscopy
NHL: Non-hodgkin lymphoma
RS: Raman spectroscopy
*H. pylori*: *Helicobacter pylori*
HPV: Human papillomavirus
MCDL: Multicentric Castleman’s disease
LANA: KSHV’s latent nuclear antigen
v-FLIP: Viral FLICE/caspase 8 inhibit protein
NGS: Next-generation sequencing
